# Medical Association of Georgia

**Published:** 1866-04

**Authors:** 


*Medical Association of Georgia.*—The last meeting held by this body was in Atlanta, on the second Wednesday in April, 1861. At that time the next annual meeting was appointed for the second Wednesday in April, 1862, in the city of Columbus. Dr. John T. Banks was elected President, Dr. A. G. Thomas, Secretary, Dr. J. F. Alexander, 1st Vice President, and Dr. V. H. Taliaferro, 2d Vice President.

We learn that no meeting has been held since 1861, and therefore, the gentlemen above mentioned are the officers of the Association now.

We think the interest of the profession requires that a meeting be held as early as practicable, and in order to this, we suggest that, it is the duty of the last elected President to appoint a meeting at the last place selected by the body, if agreeable to members of the profession there.

We do not feel content to let another year pass without renewing those pleasant associations, that in years past have proved so agreeable and profitable.

There is no doubt that by postponing the meeting a month beyond the usual time, say, to about the 15th of May, a respectable attendance could be had in Columbus, or any other place, that may be agreed upon.

While it is true that the distructive war through which we have passed, leaves physicians illy able to devote time or money to any other object than the immediate support of themselves and families, yet from the interest manifested by several physicians of our acquaintance, we feel warranted in the opinion, that the attendance will not be less than at some former meetings, in more prosperous times.

At the last meeting, Drs. O'Keefe, Taliaferro and Boyd were appointed a committee to raise $100, to be distributed for the best Essays, in the amounts of $50, $30 and $20. Drs. Coe, Means, Alexander, H. W. Brown and T. C. H. Wilson, were appointed to examine Essays.

We learn from the Chairman that the $100 for premiums will be raised.

